# The serotonin hypothesis in pulmonary hypertension revisited: targets for novel therapies (2017 Grover Conference Series)

**DOI:** 10.1177/2045894018759125

**Published:** 2018-02-22

**Authors:** Margaret (Mandy) R. MacLean

**Affiliations:** Research Institute of Cardiovascular and Medical Sciences, College of Medical, Veterinary and Life Sciences, University of Glasgow, Glasgow, UK

**Keywords:** serotonin, pulmonary hypertension, tryptophan hydroxylase 1, 5-HT1B receptor

## Abstract

Increased synthesis of serotonin and/or activity of serotonin in pulmonary arteries has been implicated in the pathobiology of pulmonary arterial hypertension (PAH). The incidence of PAH associated with diet pills such as aminorex, fenfluramine, and chlorphentermine initially led to the “serotonin hypothesis of pulmonary hypertension.” Over the last couple of decades there has been an accumulation of convincing evidence that targeting serotonin synthesis or signaling is a novel and promising approach to the development of novel therapies for PAH. Pulmonary endothelial serotonin synthesis via tryptophan hydroxlase 1 (TPH1) is increased in patients with PAH and serotonin can act in a paracrine fashion on underlying pulmonary arterial smooth muscle cells (PASMCs), In humans, serotonin can enter PASMCs via the serotonin transporter (SERT) or activate the 5-HT1B receptor; 5-HT1B activation and SERT activity cooperate to induce PASMC contraction and proliferation via activation of downstream proliferative and contractile signaling pathways. Here we will review the current status of the serotonin hypothesis and discuss potential and novel therapeutic targets.

## Introduction

The serotonin hypothesis of pulmonary hypertension (PH) was suggested in the 1990s following the observation that there was increased plasma serotonin in some patients with primary PH associated with platelet storage pool defects.^1^ In addition, diet pill-induced pulmonary arterial hypertension (PAH) was thought to be associated with the indirect serotonergic effects of aminorex, fenfluramine, and chlorphentermine.^2–7^

**Fig. 1.**
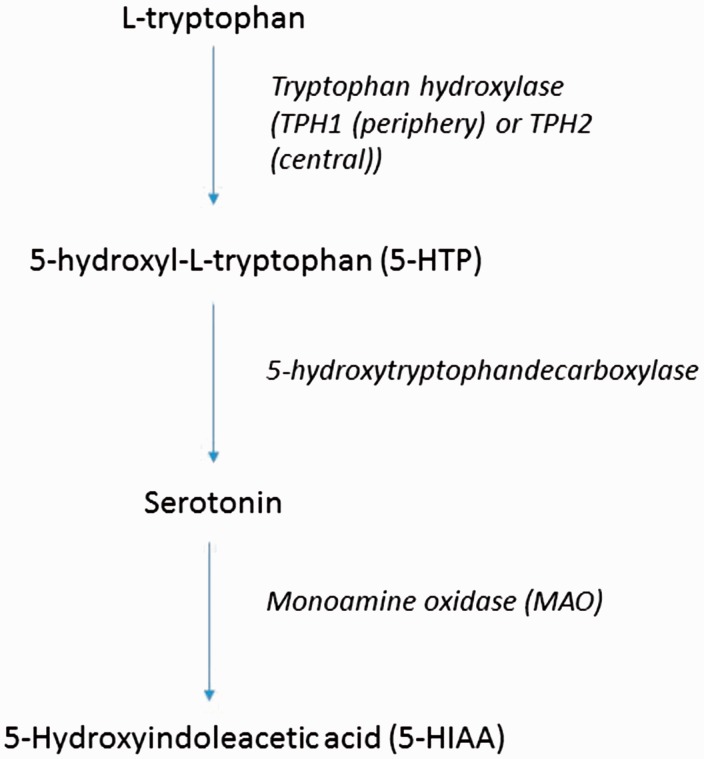
Serotonin is synthesized from L-tryptophan through the activity of TPH which converts L-tryptophan to 5-HT. This is converted to serotonin by 5-hydroxytryptophandecarboxylase and aromatic L-amino acid decarboxylase. Serotonin is metabolized to 5-HIAA via MAO and aldehyde dehydrogenase.

Serotonin is a neurotransmitter in the central nervous system and an autocoid in the periphery. It is synthesized from L-tryptophan through the activity of tryptophan hydroxylase (TPH) which converts L-tryptophan to 5-hydroxy-L-tryptophan (5-HT). This is converted to serotonin by 5-hydroxytryptophandecarboxylase and aromatic L-amino acid decarboxylase. Serotonin is metabolized to 5-hydroxyindoleacetic acid (5-HIAA) via monoamine oxidase (MAO) and aldehyde dehydrogenase (Fig. 1). The enterochromaffin cells of the gut produces 80% of the body’s serotonin; 30–80% is metabolized by the liver at first pass and 90% of the remainder is metabolized in the lung. The remaining 10% is taken up by platelets. The concentration of free serotonin in the blood is therefore normally extremely low. Indeed, carefully controlled studies in patients devoid of platelet storage pool disease have failed to demonstrate an increase in free serotonin in the blood of patients with PAH.^8^

**Fig. 2.**
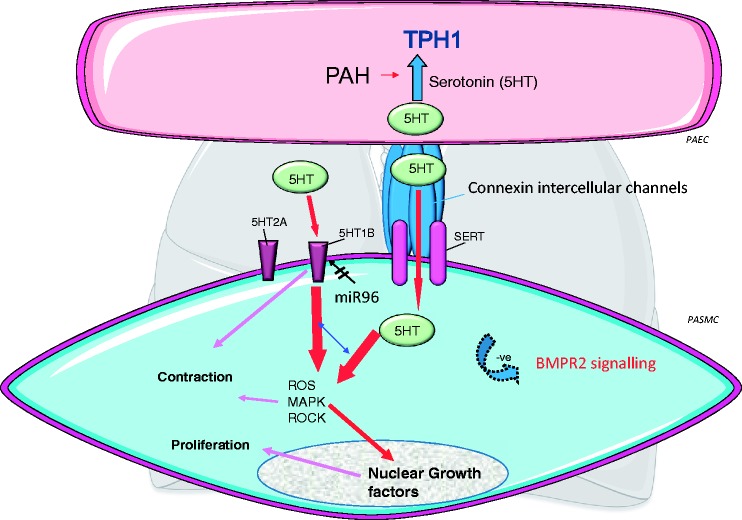
Serotonin synthesis via tryptophan hydroxylase 1 (TPH1) is increased in pulmonary artery endothelial cells (PAECs) from rodent models of PH (inset showing a small pulmonary artery from a control and hypoxic rat with TPH1 staining in the PAECs) and patients with PAH. Serotonin can act in a paracrine fashion on underlying PASMCs, facilitated by myoendothelial gap junctions (connexion intercellular channels). Serotonin can enter the PASMC via the Serotonin transporter (SERT) or activate serotonin receptors. The important receptor in the human pulmonary arterial smooth muscle cell (PASMC) is the 5-HT1B receptor, regulated by microRNA96 (miR96) such that it is upregulated (by decreased miR96 expression) in female PAH patient PASMCs. 5-HT1B activation and SERT activity cooperate to induce PASMC contraction and proliferation via increased ROS and activation of downstream signaling pathways such as MAPK and rho-kinase (ROCK). These can also facilitate nuclear growth factors such as GATA-4. Increased serotonin can facilitate a pulmonary hypertensive phenotype in BMPR2-/+ mice via decreased BMPR2 signaling.

Since the 1990s, researchers have been interrogating the serotonin system in the pulmonary circulation and a summary of some of these studies is shown in Fig. 2.

We now know that in patients with PAH, pulmonary arterial endothelial TPH1 expression is increased and that endothelial-derived serotonin can act on the underlying pulmonary arterial smooth muscle cells (PASMCs) in a paracrine fashion.^9^ Recently, this has been shown to be facilitated by myoendothelial gap junctions.^10^ Endothelial TPH1 expression is also increased in animal models of PH.^11^ Pathologically, PAH is characterized by vasoconstriction of the small pulmonary arteries and proliferation in all layers of the vessel wall as well as fibrosis and inflammation. Therefore, serotonin may have many pathological influences on the pulmonary arterial circulation. In PASMCs, it can activate serotonin receptors to induce proliferation and contraction,^12–16^ inhibit voltage-gated K+ currents which would elevate vascular tone,^17^ or enter the cell via the serotonin transporter (SERT). Serotonin can subsequently activate mitogen-activated tyrosine kinases via superoxide production.^18–20^ It can induce Rho kinase-induced nuclear translocation of ERK1/ERK2 to cause mitogenesis.^18–21^ Serotonin can increase the susceptibility of BMPR2+/− mice to hypoxia-induced PH. PASMCs from BMPR2(+/−) mice exhibited a heightened DNA synthesis and activation of extracellular signal-regulated kinase 1/2 in response to serotonin compared with wild-type cells. Serotonin inhibits BMP signaling via Smad proteins and the expression of BMP responsive genes.^22^

Transglutaminase 2 (TG2) is a multifunctional enzyme that cross-links proteins with monoamines such as serotonin via a transglutamidation reaction (serotonylation), and is associated with pathophysiologic vascular responses.^23,24^ Through these mechanisms, serotonin can induce PASMC proliferation and contraction. RhoA serotonylation, following SERT-mediated cellular internalization of serotonin has been described in platelets.^25^ RhoA and Rho kinase activities are increased in idiopathic PAH (iPAH), in association with enhanced RhoA serotonylation and this may involve platelet activation.^26^ Serotonin-induced fibrosis may also play a role in PAH. Serotonin can activate pulmonary arterial fibroblasts and promote adventitia fibrosis through signaling of the TGFβ1/Smad3 pathway^27^ and, in PASMCs, through NADPH oxidase (Nox)1.^28^ An interplay between PDGF and serotonin pathways within PAH has also been demonstrated to explain TPH1-dependent imatinib efficacy in collagen-mediated mechanisms of fibrosis.^29^ Serotonin also activates immune responses and inflammation in many peripheral diseases^30^ and these effects may also play a role in the development of PAH.

In order to consider if the serotonin system may provide a therapeutic target for PAH, we need to consider its activation of receptors, the SERT, and its synthesis via TPH.

## Serotonin receptors in the pulmonary circulation

There are three major subtypes of 5-HT receptors: 5-HT_1_; 5-HT_2_; and 5-HT_3_; which exist in flies, molluscs, round worms, rodents, rabbits, cats, dogs, and humans.^31^ The International Union of Pharmacology classification of 5-HT receptors further divides these into 5-HT_1A_, 5-HT_1B_, etc.^32^ Other 5-HT receptors are suggested by their cDNAs but still need to be confirmed.

5-HT2A receptors predominate in systemic arterial medial tissue and are expressed in the normal PASM of species such as rats and mice but it is the 5-HT1B receptor that normally mediates pulmonary arterial responses to serotonin in larger animals and man.^14,33–37^ Antagonism of the 5-HT2B receptor has been shown to have therapeutic effects in rodent models of PH^38–40^ and in mice, activation of 5-HT2B receptors on bone marrow lineage progenitors is critical for the development of experimental PH.^41^ A role for the 5-HT2B receptor in human PAH has yet, however, to be established. Indeed, a mutation in the 5-HT2B receptor has been demonstrated in a patient with PAH^42^ and the selectivity of potential 5-HT2B antagonists for 5-HT2B versus 5-HT1B receptors are unclear. It is still unclear if the 5-HT2B receptor will prove to be a good target for human PAH.

### The 5-HT1B receptor

In 1993, adverse reactions in patients taking the 5-HT1B/D agonist sumatriptan for migraine led to the discovery that this was due to pulmonary vasoconstriction via the 5-HT1B receptor.^34,35^ The 5-HT1B receptors are negatively linked to adenylate cyclase and suppress forskolin-stimulated cyclic AMP accumulation, stimulating increases in [Ca2+]i,^43^ thus causing vasoconstriction. What is very relevant to the influence of the 5-HT1B receptor in the pulmonary artery is that it can amplify the accumulation of [3H] inositol phosphates elicited by a Gq-protein coupled receptor.^43^ In addition, removal of the vascular endothelium, inhibition of nitric oxide synthesis, and small increases in vascular tone synergize such that they hugely amplify the effects of 5-HT1B activation in pulmonary arteries.^13,36,44–46^ As pulmonary arterial endothelial dysfunction, nitric oxide synthesis and increased vascular tone are all contributing factors in PAH, this increases the relevance of the 5-HT1B receptor in this disease. Pulmonary arterial responses to the 5-HT1B receptor are amplified in experimental PH^13,45^ and ablation or antagonism of the receptor can reverse experimental PH.^47^ Curiously, Raynaud’s phenomenon is correlated with increased pulmonary arterial pressures in patients with lupus.^48^ This may be driven by the 5-HT1B receptor and variants in the 5-HT1B gene have been shown to be associated with Raynaud’s.

Recently, it was shown that hPASMCs from female PAH patients over-express the 5-HT1B receptor and these mediate serotonin-induced proliferation in these cells. The increased 5-HT1B receptor expression may be a consequence of decreased microRNA-96 expression in the female patient PASMCs, mediated by estrogen, and this may contribute to the development of PAH.^49^ Restoration of miRNA-96 expression in pulmonary arteries in vivo via administration of an miRNA-96 mimic reduced the development of hypoxia-induced PH in the mouse.^49^ The 5-HT1B receptor can mediate proliferation, vasoconstriction, and fibrosis in the human pulmonary circulation^14,16,35^ and in animal models of PH.^13,36,45,50,51^ Antagonism or knockout of the 5-HT1B receptor can also ablate experimental PH.^47,52^ The pulmonary vascular injury induced by 5-HT1B activation may be due to oxidative stress caused by Nox1 activation combined with Nrf-2 dysfunction.^52^

## Serotonin and oxidative stress

Increased bioavailability of reactive oxygen species (ROS; superoxide anion and hydrogen peroxide) can lead to oxidative stress which has been associate with experimental and human PH by many groups over the years.^53–59^ Serotonin is known to induce hydrogen peroxide by activating Nox or monoamine oxidase (MAO)-A. Metabolism by MAO plays an incremental, predominant role in biogenic amine turnover, which may lead to greater oxidative stress. Oxidation of monoamine substrates, particularly by MAO-B, increases the generation of ROS and this may play a role in chronic neurodegenerative processes, particularly in the central nervous system.^41,60^ Iproniazid (a MAO-A inhibitor) can block serotonin-induced S100A4/Mts1 in hPASMCs,^12^ but a definitive role for MAO in the development of PH is as yet unclear.

Hydrogen peroxide can phosphorylate ERK and/or activate Rho kinase facilitating ERK translocation. ERK can thereby phosphorylate nuclear growth factors such as GATA4.^12,19,21,61^ Cellular production of antioxidants are frequently regulated by nuclear factor erythroid-related factor 2 (Nrf2), which is a transcription factor that influences antioxidant genes such as superoxide dismutase, catalase, and thioredoxin, all of which protect against oxidative damage.^62^ We recently demonstrated that the pulmonary vascular injury induced by 5-HT1B activation in hPASMCs may be due to oxidative stress caused by Nox1 activation and subsequent ROS production combined with Nrf-2 dysfunction.^52^ Indeed, the Nrf2 activator bardoxolone is being considered for use in PAH.^63^ One of the most important consequences of oxidative stress is oxidation of proteins, particularly redox-sensitive PTPs, which regulate phosphorylation of downstream proteins, including mitogen-activated protein kinases (MAPK), such as p38MAPK, and we have shown that serotonin can increase irreversible PTP oxidation in PAH-hPASMCs in a 5-HT1B-dependent fashion. Serotonin also induces 5-HT1B dependent Rho-kinase activation in these cells.^52^ E2, through ERα, can also increase Nox-derived ROS and redox-sensitive growth in hPASMCs, with greater effects in cells from PAH patients. These effects may be via conversion of E2 to 16α-OHE1 by CYP1B1.^28^

## The serotonin transporter

The serotonin transporter (SERT or 5-HTT) is a monoamine transporter protein that transports serotonin into cells. This transport of serotonin by the SERT recycles it in a sodium-dependent manner. The SERT may be over-expressed via a SERT gene polymorphism in certain cohorts of patients with PAH;^64,65^ however, analysis of other cohorts of patients were unable to confirm this.^66,67^ The hypothesis is that hPASMC proliferation is mediated via SERT activity.^64^ In mice, ubiquitous over-expression of SERT causes the development of PH in female mice.^68^ In mice (both male and female), over-expression of SERT in the smooth muscle also induces PH.^69^ There is attenuated hypoxia-induced PH in SERT gene knockout mice.^70^ Inhibition of SERT protects against hypoxia-induced PH.^71^ Further studies of SERT over-expressing mice demonstrated that the PH phenotype was only observed in females aged 5–6 months and was related to estrogen and associated with over-expression of CYP1B1, an enzyme important in the metabolism of estrogen.^72,73^

Dexfenfluramine is an indirect serotinergic agonist, in that it can enter cells via the SERT and induce release of serotonin. As such, it can compete with serotonin for the SERT. There may be therefore be synergistic effects between dexfenfluramine and SERT in inducing pulmonary vascular remodeling in hypoxic mice. On its own, dexfenfuramine can inhibit hypoxia-induced pulmonary vascular remodeling via SERT activity.^74^ It is unlikely that SERT inhibitors (SSRIs) would be effective in PAH as they actually increase pulmonary vascular contraction due to causing extra-cellular accumulation of serotonin and subsequent 5-HT1B receptor activation.^75^ Indeed, the use of antidepressants has been associated with a non-causal but significantly increased risk of iPAH.^76^ In addition, in a large population of patients with PAH, SSRI use was associated with increased mortality and a greater risk of clinical worsening.^77^ There are several studies, however, that suggest that the 5-HT1B receptor and SERT cooperate in both the contractile and proliferative effects of serotonin in the pulmonary circulation.^12,50,51,75^ The fawn-hooded rat has been studied as a model of human PAH because it has altered serotonergic function, an inherited platelet pool storage defect to serotonin, increased circulating levels of serotonin, and increased pulmonary vascular responsiveness to serotonin.^78–80^ Indeed, SERT inhibitors may increase pulmonary vasoconstriction in this rat model of PAH and this can be inhibited by simultaneous 5-HT1B receptor antagonism.^75^

Heightened expression of the S100 calcium-binding protein, S100A4/Mts1, is observed in pulmonary vascular disease and there is a mechanistic link between the serotonin pathway and S100A4/Mts1 in hPASMCs where the 5-HT1B receptor and SERT are co-dependent in regulating S100A4/Mts1.^12^ A combined 5-HT1B receptor antagonist and SERT inhibitor was more effective than a SERT inhibitor alone at reversing serotonin-induced proliferation in IPAH hPASMCs.^51^ There is also synergy between 5-HT1B receptor and serotonin transporter inhibitors against serotonin-induced vasoconstriction in mouse and rat pulmonary arteries.^51,75^ Evidence therefore suggests that targeting both the serotonin transporter and the 5-HT1B receptor may be a novel therapeutic approach to PAH.

## TPH1

Tryptophan hydroxylase (TPH) catalyzes the rate limiting step in serotonin synthesis. TPH1 is expressed in the gut, pineal gland, spleen, and thymus and is responsible for the synthesis of peripheral serotonin while TPH2 is predominantly expressed in the brain stem and is responsible for the synthesis of central serotonin.^81,82^ In the periphery, serotonin has effects on the immune system, the gastrointestinal tract, hemostasis, melatonin synthesis, vasoconstriction, and cellular proliferation. In the central nervous system, it has effects on anxiety, nerve activity, mood, food intake, aggression, and sleep. In both patients with PH and in animal models of PH, pulmonary endothelial TPH1 expression is increased.^9,11^ There is growing evidence that genetic ablation of TPH1 or pharmacological inhibition can protect against or reverse experimental PH including in the hypoxic, monocrotaline, dexfenfluramine, and sugen/hypoxic animal models.^11,29,74,83–86^ This has led to recent interest in the use of TPH1 inhibitors for the treatment of PAH patients. Post hoc subgroup analysis has suggested that PAH patients with greater hemodynamic impairment had reduced serotonin plasma levels following imatinib treatment, compared with placebo. This study suggested that imatinib may downregulate TPH1 via inhibition of PDGFβ signaling.^29^ To date, drug design for TPH1 selectivity has been dependent on developing unselective TPH inhibitors that do not cross the blood–brain barrier.^87^ However, biochemical and biophysical characterization of a novel allosteric site on TPH1 through which selectivity over TPH2 and related aromatic amino acid hydroxylases can be achieved has recently been described.^88^ This should enable the development of TPH1 selective drugs.

## Serotonin and BMPR2

The primary genetic defect of heritable PAH (hPAH) is a mutation in the gene encoding BMPR2 (present in at least 70–80% of cases of hPAH and >25% of iPAH). hPAH transmits as an autosomal dominant trait that exhibits genetic anticipation but also markedly reduced penetrance.^89–91^ Females with BMPR2 mutations have a higher disease penetrance and are about 2.5-fold more likely to develop hPAH than males.^91^ The cause of the reduced penetrance is likely to be related to a “second hit” caused by environmental and/or genetic modifiers. BMPR2 is a member of the transforming growth factor beta (TGF-β) superfamily. Signaling by BMP-receptors involves heterodimerization of two transmembrane serine/threonine kinases: the constitutively active type 2-receptor, BMPR2, and a corresponding type 1-receptor, BMPR1A or BMPR1B. Activated BMPR1-receptors phosphorylate a set of BMP restricted Smad proteins, (Smad1, 5, and 8), which then complex with the common partner Smad, Smad4 (Co-Smad), and translocate into the nucleus to regulate transcription of target genes. Inhibitor of DNA binding (Id) family of protein (Id1–4) are transcriptional targets of BMP signaling and bind with high affinity to the E protein family of basic-loop-helix family of transcription factors and inhibit their binding to target DNA, regulating gene expression and cellular differentiation. Dysfunctional Smad signaling leads to abnormal cell proliferation associated with pulmonary vascular disease.^92^

### Synergy between serotonin and BMPR2

We have previously shown that serotonin uncovers a PH phenotype in BMPR2+/− mice suggesting that serotonin may be a required second hit facilitating the pathogenic effects of BMPR2 happloinsufficiency.^22^ On its own, infused serotonin did not induce a PH phenotype. This suggests that it is a local, pulmonary endothelial released serotonin acting directly on PASMCs with a BMPR2 dysfunction that drives the pathogenesis of PH. This is consistent with fact that elevated circulating serotonin levels on their own (for example, as observed in carcinoids syndrome) rarely cause PAH. We also examined miRNA-96 expression in PASMCs from female BMPR2 (R899X+/−) mice; we demonstrated that the proliferative response to serotonin increased, associated with increased 5-HT1BR expression and a decrease in miRNA-96.

### Sex, serotonin, and BMPR2

It is only the female BMPR2 (R899X+/−) mice (unpublished) and Smad1-/- mice^93^ that spontaneously develop PAH. In the BMPR2 (R899X+/−) mice, this is via 5-HT1B-mediated effects regulated by estrogen via inhibition of miRNA-96.^49^ We have also previously shown that inhibition of endogenous synthesis of E2 with the aromatase inhibitor anastrozole, reverses hypoxic- and sugen/hypoxic-induced PH in rats but only in female rats. The female rodent lung displays increased aromatase and decreased BMPR2 and Id1 expression compared with the male lung. Anastrozole treatment reversed the impaired BMPR2 pathway in females only.^94^ We have also demonstrated that E2-driven suppression of BMPR2 signaling in non-PAH hPASMCs derived from women contributes to a pro-proliferative phenotype in hPASMCs that may predispose women to PAH.^93^ E2 also increases expression of TPH1 and the 5-HT1BR in hPASMCs.^73^ In humans, any treatment that targets endogenous E2 will, however, be as effective in males as females because endogenous PASM expression of aromatase is observed in female PAH patients pre and post menopause and in male patients also.^94^ Plasma E2 levels are also elevated in male iPAH patients.^95^

## Measuring serotonin

There are several ways of measuring serotonin in the blood and urine, including HPLC (electrochemical and fluorometric), radioenzymatic assay, immunoassay (e.g. ELISA), spectrophotofluorometric, GC-MS, and liquid chromatography-tandem mass spectrometry. Measuring free serotonin levels in the plasma is, however, problematic and the actual concentration reported can be in the range of 0.6–180 nmol/L depending on the methods used.^96^ There are many other factors that need considered when evaluating serotonin concentrations. Serotonin- or tryptophan-rich foods such as dates, grapefruit, cantaloupe, avocados, bananas, plums, eggplant, plantain, walnuts, pineapple, tomatoes, hickory nuts, kiwi, or honeydew melon can increase urinary serotonin and urinary 5-HIAA levels markedly. Drugs such as lithium, MAO inhibitors, methyldopa, and morphine all elevate urine and serum serotonin. SSRIs can lead to depletion of platelet serotonin levels and result in false-negative urine, serum, and blood serotonin tests. Heavy nicotine consumption via heavy smoking can also result in false elevations of urinary serotonin levels as there can be cross-reactivity of the major nicotine metabolite cotinine with serotonin in some assays.

## Summary

PAH is a very complex disease and many factors are involved in its pathophysiology. There is a wealth of evidence in support of the “serotonin hypothesis of PAH” and that serotonin is one factor contributing to the development of PAH. In light of this, it is hoped that future direction sees the development of inhibitors of either serotonin synthesis (TPH1 inhibitors) or the 5-HT1B receptor which may prove to be therapeutically effective in PAH.

### 2017 Grover Conference Series

This review article is part of the 2017 Grover Conference Series. The American Thoracic Society and the conference organizing committee gratefully acknowledge the educational grants provided for the support of this conference by Actelion Pharmaceuticals US, Inc., Gilead Sciences, Inc., and United Therapeutics Corporation. Additionally, the American Thoracic Society is grateful for the support of the Grover Conference by the American Heart Association, the Cardiovascular Medical Research and Education Fund, and the National Institutes of Health.

### 2017 PVRI Annual Drug Discovery & Development Symposium

This review article is also based on a presentation given by the author at the 4th Annual Drug Discovery & Development Symposium for Pulmonary Hypertension in Berlin.
